# P-382. Characteristics and Outcomes of People with HIV (PWH) with Suboptimal Adherence on B/F/TAF or Dolutegravir Single-Tablet Regimens (STRs)

**DOI:** 10.1093/ofid/ofaf695.600

**Published:** 2026-01-11

**Authors:** Richard A Elion, Joshua Gruber, Megan Dunbar, Janna Radtchenko, Neia Prata Menezes, Keith Dunn, Travis Lim, Charles M Walworth, Joseph J Eron, Paul E Sax, Steven Santiago, Moti Ramgopal

**Affiliations:** Trio Health, Louisville, Colorado; Gilead Sciences, Forest City, California; Gilead Sciences, Forest City, California; Trio Health, Louisville, Colorado; Gilead Sciences, Inc., Foster City, California; Gilead Sciences, Forest City, California; Gilead Sciences, Inc., Foster City, California; Monogram Biosciences/LabCorp, Laguna Beach, CA; University of North Carolina at Chapel Hill School of Medicine, Chapel Hill, North Carolina; Brigham and Women’s Hospital; Harvard Medical School, Boston, MA; CareResource, Miami, Florida; Midway Immunology and Research Center, Fort Pierce, Florida

## Abstract

**Background:**

INSTI-based regimens are recommended for PWH with suboptimal adherence [SA] due to their forgiveness and high genetic barrier to resistance. We characterized adherence and persistence among PWH receiving B/F/TAF or DTG STRs and evaluated virologic outcomes in PWH with SA.

**Table 1.**
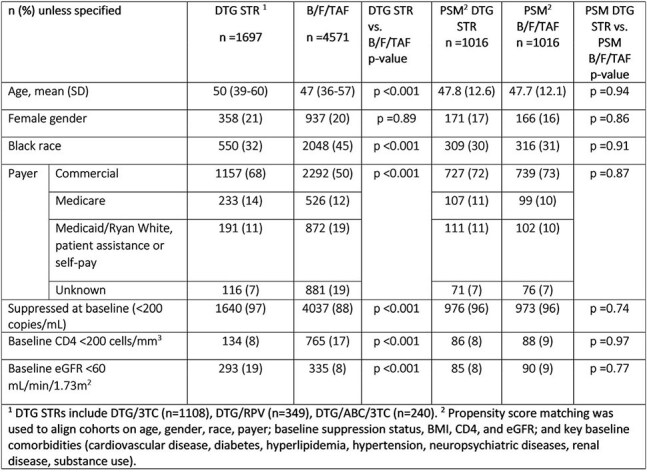
Baseline Characteristics

**Table 2.**
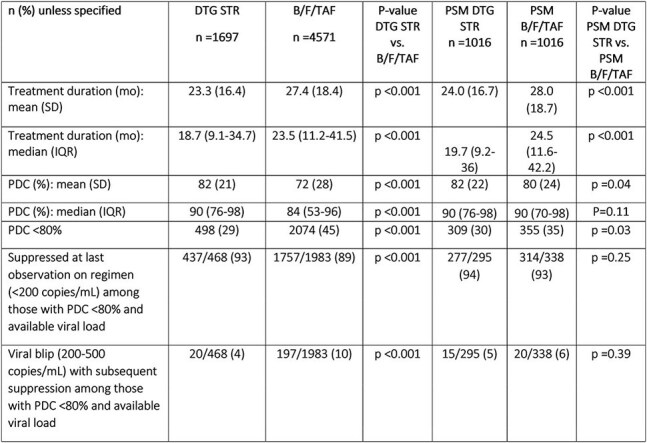
Outcomes

**Methods:**

Retrospective study using Trio Health EMR and dispensing data (04/2019-09/2024). Inclusion: PWH age ≥ 18 indexed at switch to B/F/TAF or DTG STR, with ≥ 2 dispenses and ≥ 3 months on index regimen, and ≥ 1 years of follow-up. Outcomes: persistence (mean/median regimen duration), adherence (mean/median proportion days covered [PDC], proportion with SA: PDC < 80%); if with SA, viral suppression (viral load [VL] < 200 copies/mL at last observation), viral blips [VB] (VL 200-500 copies/mL with suppression). Propensity score matching (PSM) balanced B/F/TAF and DTG STR groups by baseline characteristics.

**Results:**

Differences in baseline characteristics were observed between 4571 PWH on B/F/TAF and 1697 on DTG STR [Table 1]. Among DTG STR, 1108 (65%) were on DTG/3TC, 349 (21%) on DTG/RPV, and 240 (14%) on DTG/ABC/3TC. Persistence was higher on B/F/TAF (median/mean 24.5/27.4 months) vs. DTG STRs (median/mean 19.7/23.3 months). Median/mean PDC was 84%/72% for B/F/TAF and 90%/82% for DTG STR, with 45% and 29% with SA, respectively [Table 2]. Among PWH with SA, 89% on B/F/TAF and 93% on DTG STRs were suppressed at last observation. After PSM, suppression was equally high across regimens: 93% for B/F/TAF vs. 94% for DTG STRs. Among those with SA, VB were more frequent for B/F/TAF (10%) vs. DTG STRs (4%) before PSM, but not after (6% B/F/TAF vs. 5% DTG STR).

**Conclusion:**

We observed higher persistence on B/F/TAF and higher adherence on DTG STRs. After PSM, PWH with SA had similar virologic outcomes across regimens. Findings suggest that B/F/TAF and DTG STRs are effective ARTs for PWH in need of additional adherence support.

**Disclosures:**

Richard A. Elion, MD, Gilead Sciences: Advisor/Consultant|Gilead Sciences: Grant/Research Support|Trio Health: Employee|ViiV Healthcare: Advisor/Consultant|ViiV Healthcare: Grant/Research Support Joshua Gruber, PhD MPH, Gilead Sciences: Employee and shareholder Megan Dunbar, PhD, Gilead Sciences, Inc.: Employee|Gilead Sciences, Inc.: Stocks/Bonds (Public Company) Janna Radtchenko, MBA, Trio Health: Employee Neia Prata Menezes, PhD, Gilead Sciences, Inc.: Stocks/Bonds (Private Company) Keith Dunn, PharmD, Gilead Sciences: Employee|Gilead Sciences: Stocks/Bonds (Public Company) Travis Lim, MSc, DrPH, Gilead Sciences, Inc.: Employee|Gilead Sciences, Inc.: Stocks/Bonds (Public Company) Charles M. Walworth, MD, Labcorp: Employee|Labcorp: Stocks/Bonds (Public Company)|Labcorp: Stocks/Bonds (Public Company) Joseph J. Eron, MD, Gilead Sciences: Advisor/Consultant|Gilead Sciences: Grant/Research Support|Merck: Advisor/Consultant|Merck: Grant/Research Support|ViiV Healthcare: Advisor/Consultant|ViiV Healthcare: Grant/Research Support Paul E. Sax, MD, Gilead Sciences: Advisor/Consultant|Gilead Sciences: Grant/Research Support|Gilead Sciences: Honoraria|Merck: Advisor/Consultant|Merck: Grant/Research Support|ViiV Healthcare: Advisor/Consultant|ViiV Healthcare: Grant/Research Support Steven Santiago, MD, Gilead Sciences: Advisor/Consultant Moti Ramgopal, MD, AbbVie: Honoraria|Gilead Sciences, Inc.: Advisor/Consultant|Gilead Sciences, Inc.: Honoraria|Shionogi: Advisor/Consultant|ViiV: Advisor/Consultant|ViiV: Honoraria

